# Mutation of *praR* in *Rhizobium leguminosarum* enhances root biofilms, improving nodulation competitiveness by increased expression of attachment proteins

**DOI:** 10.1111/mmi.12670

**Published:** 2014-07-02

**Authors:** Marijke Frederix, Anne Edwards, Anna Swiderska, Andrew Stanger, Ramakrishnan Karunakaran, Alan Williams, Pamela Abbruscato, Maria Sanchez-Contreras, Philip S Poole, J Allan Downie

**Affiliations:** Department of Molecular Microbiology, John Innes CentreNorwich Research Park, Norwich, NR4 7UH, UK

## Abstract

In *Rhizobium leguminosarum* bv. *viciae*, quorum-sensing is regulated by CinR, which induces the *cinIS* operon. CinI synthesizes an AHL, whereas CinS inactivates PraR, a repressor. Mutation of *praR* enhanced biofilms *in vitro*. We developed a light (*lux*)-dependent assay of rhizobial attachment to roots and demonstrated that mutation of *praR* increased biofilms on pea roots. The *praR* mutant out-competed wild-type for infection of pea nodules in mixed inoculations. Analysis of gene expression by microarrays and promoter fusions revealed that PraR represses its own transcription and mutation of *praR* increased expression of several genes including those encoding secreted proteins (the adhesins RapA2, RapB and RapC, two cadherins and the glycanase PlyB), the polysaccharide regulator RosR, and another protein similar to PraR. PraR bound to the promoters of several of these genes indicating direct repression. Mutations in *rapA2*, *rapB*, *rapC*, *plyB*, the cadherins or *rosR* did not affect the enhanced root attachment or nodule competitiveness of the *praR* mutant. However combinations of mutations in *rapA*, *rapB* and *rapC* abolished the enhanced attachment and nodule competitiveness. We conclude that relief of PraR-mediated repression determines a lifestyle switch allowing the expression of genes that are important for biofilm formation on roots and the subsequent initiation of infection of legume roots.

## Introduction

The infection of legume roots by rhizobia, leading to the formation of nitrogen-fixing nodules, is a clonal event and each individual bacterium that initiates an infection can grow rapidly, giving rise to over 10^6^ progeny in nodules. It is somewhat of a lottery which individual soil *Rhizobium* will succeed in infecting roots and nodules, but critical to success is the ability of rhizobia to attach to legume roots and root hairs at potential infection sites (Downie, 2010). This attachment involves the secretion of both proteins that act as adhesins and different surface polysaccharides (Milner *et al*., 1992; Bittinger *et al*., 1997; Pobigaylo *et al*., 2008; Williams *et al*., 2008; Janczarek *et al*., 2009). Rhizobia attached to roots and root hairs are in an ideal position to detect plant-made signals such as flavonoids, which induce the production of nodulation signals (Nod-factors), that induce plant programmes for initiating infection and stimulating nodule morphogenesis (Oldroyd and Downie, 2008).

In view of the selective advantages of growing on exudates from root hairs and from infecting nodules, it is not surprising that rhizobia have multiple ways of attaching to roots and root hairs. This can involve different surface polysaccharides, secreted proteins and production of Nod-factor signalling molecules (Downie, 2010). Under slightly acidic conditions, a plant lectin expressed on root-hair tips promotes attachment of *Rhizobium leguminosarum* biovar *viciae* (*R.l. viciae*) to pea root hairs by binding to a polar-localized bacterial surface polysaccharide called glucomannan (Laus *et al*., 2006). Blocking glucomannan synthesis by the *gmsA* mutation did not block nodulation, but greatly reduced the ability of the mutants to compete with WT for nodule infection in competitive nodulation tests (Williams *et al*., 2008). Under slightly alkaline conditions, the lectin-glucomannan-mediated attachment is poor and a secreted calcium-binding protein called rhicadhesin becomes the dominant mechanism of attachment (Smit *et al*., 1987; 1989; Laus *et al*., 2006). The gene encoding rhicadhesin has not been identified, but a search for secreted proteins that attach to the surface of *R. leguminosarum* identified a group of rhizobial adhering proteins (Raps) that promote attachment and aggregation by rhizobia (Ausmees *et al*., 2001). These Rap proteins are calcium-binding lectins containing cadherin-like domains that bind to the acidic exopolysaccharide (Abdian *et al*., 2013), which is essential for attachment both *in vitro* and to root hairs (Russo *et al*., 2006; Williams *et al*., 2008). Increased expression of one of these proteins, RapA1, enhanced attachment to roots and increased infection competitiveness (Mongiardini *et al*., 2008; 2009). Cellulose fibrils play a role in biofilm growth (called cap formation) on root hairs after initial attachment, although a mutant unable to produce cellulose nodulated normally and apparently was not reduced for nodulation competitiveness (Smit *et al*., 1987; Laus *et al*., 2005; Williams *et al*., 2008). There are also other plant components such as an arabinogalactan protein, which can influence the attachment of *R. leguminosarum* to surfaces (Xie *et al*., 2012).

In previous work, we observed that mutations affecting the *cinR–cinIS* encoded quorum-sensing regulatory system affected biofilm formation *in vitro* (Edwards *et al*., 2009; Frederix *et al*., 2011). The *cinS* gene, co-transcribed with the AHL synthase gene *cinI*, encodes a small protein that acts as an anti-repressor of the transcriptional regulator PraR (Edwards *et al*., 2009; Frederix *et al*., 2011). In that work, PraR was identified because it bound to CinS, but PraR is highly conserved among the α proteobacteria (Akiba *et al*., 2010). PraR belongs to the HipB family of DNA-binding proteins and contains an N-terminal (residues 19–73) cro/C1-like helix–turn–helix domain and probably has a multimerization domain based on its pattern of binding to DNA (Frederix *et al*., 2011). In *Escherichia coli* HipB regulates the *hipBA* toxin-antitoxin operon (Gerdes and Maisonneuve, 2012); also belonging to this family is SinR a master regulator of *Bacillus subtilis* biofilms (Kearns *et al*., 2005). Repression mediated by HipB and SinR is relieved by their binding to the antirepressors HipA and SinI respectively. SinR has a terameric structure and in solution SinI destabilizes this tetramer by tightly binding individual SinR subunits forming a 1:1 dead-end complex, thereby reducing the ability of SinR to bind to DNA (Scott *et al*., 1999). We assume CinS destabilizes PraR in a similar manner, and indeed CinS is stabilized in the presence of PraR suggesting that a stable CinS-PraR complex is formed (Frederix *et al*., 2011).

In *Azorhizobium caulinodans*, PraR represses the expression of *reb* genes that are present in *A. caulinodans*, but absent from most rhizobia; the increased expression of the *reb* genes in a *praR* mutant caused decreased nitrogen fixation in nodules (Akiba *et al*., 2010). PraR is orthologous to PhrR from *Sinorhizobium medicae*, which was originally identified as a transcriptional regulator induced by low pH (Reeve *et al*., 1998), but was named *praR* in A. *caulinodans* and *R. leguminosarum* because induction by low pH was not observed in those strains (Akiba *et al*., 2010; Frederix *et al*., 2011).

In *R.l. viciae*, PraR represses the *raiR* and *rhiR* genes, which encode different LuxR-type quorum-sensing regulators. This repression is relieved as the population density increases and *cinS* expression is increased, and so the antirepressor CinS binds to soluble PraR relieving PraR-mediated repression (Edwards *et al*., 2009; Frederix *et al*., 2011). When induced by relief of repression, RhiR induces the expression of the AHL synthase encoded by *rhiI* and the consequent population-dependent induction of *rhiABC* genes plays a role in rhizosphere growth and nodulation (Cubo *et al*., 1992). The other regulator RaiR regulates the expression of the AHL synthase gene *raiI*, and in some strains, mutation of *raiI* increased nitrogen fixation in nodules (Rosemeyer *et al*., 1998). Not many strains have the *raiI* and *raiR* genes and genes regulated via the *raiIR* quorum-sensing system have not been identified.

In addition to directly repressing *rhiR* and *raiR* (Frederix *et al*., 2011), PraR may repress the expression of *plyB* in *R.l. viciae;* PlyB is one of three secreted polysaccharide glycanases that cleave the surface EPS, and the pattern of *plyB* expression mirrored that of *raiR* and *rhiR* in various quorum-sensing mutants (Edwards *et al*., 2009; Frederix *et al*., 2011). However, apart from the *rhiR* and *raiR* promoters, no other direct targets of PraR have been demonstrated.

In this work, we used microarray analysis, promoter gene fusions and promoter binding experiments to identify direct targets of PraR in *R.l. viciae*. Among the genes directly repressed by PraR, are *praR*, three *rap* genes, *rosR*, encoding a global regulator of polysaccharides and one gene similar to *praR*. We demonstrate that mutation of *praR* results in enhanced *in vitro* biofilm formation, enhanced attachment to root hairs and increased nodulation competitiveness primarily due to enhanced expression of Rap proteins.

## Results

### Mutation of *praR* increases biofilms and expression of genes encoding secreted attachment proteins

**Fig. 1:**
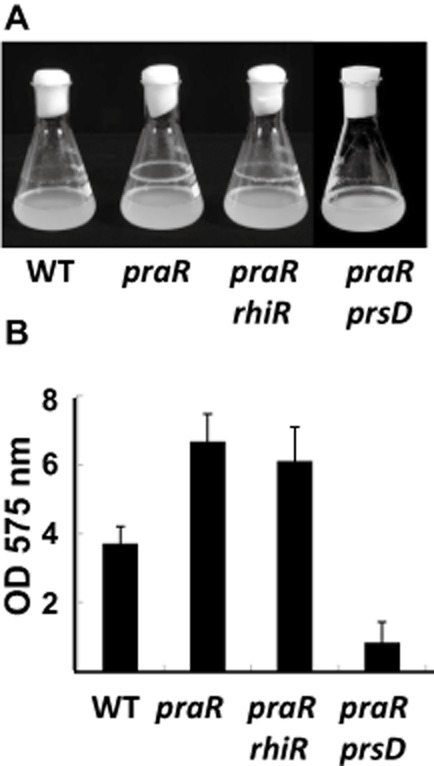
Mutation of *praR* enhances biofilm formation by *R.l. viciae.*A. Formation of rings of biofilm at the air–liquid interface after 5-day growth of 3841 (WT) and the mutants A963 (*praR*), A1149 (*praR, rhiR*) and A1161 (*praR, prsD*) in Y mannitol medium.B. The biofilms were quantified following staining with crystal violet and the data shown are averages (*n* = 10) ± SD. The mutants are all significantly different from WT (*P* = < 0.05 Student's *t*-test), whereas the *praR*, *rhiR* double mutant is not significantly different from the *praR* mutant.

Mutation of *praR* in *R.l. viciae* strain 3841 enhanced biofilm formation both in polystyrene microtitre dishes (data not shown) and at the air–liquid interface on glass shake flasks (Fig. 1A). Quantification of the attachment using crystal violet staining confirmed that there was an increased biofilm with the *praR* mutant compared with WT (Fig. 1B). Since mutation of *praR* increased expression of *rhiR* (Frederix *et al*., 2011), we tested whether the increased biofilms were due to quorum sensing regulated by RhiR, by introducing a *rhiR* mutation into the *praR* mutant. Since the double mutant retained an enhanced biofilm (Fig. 1A and B), RhiR was not required. Strain 3841 lacks *raiI* and *raiR* and so the *praR* mutant phenotype could not be mediated via RaiR.

**Fig. 2:**
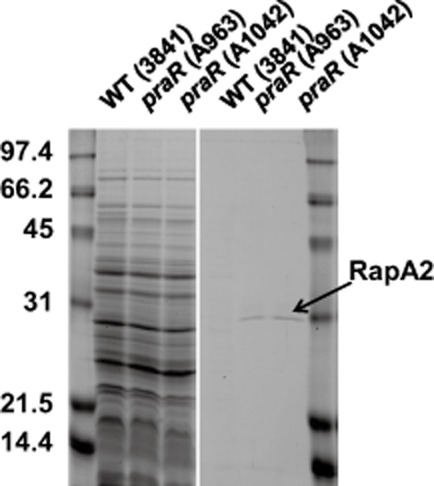
Mutation of *praR* enhances the level of the *R.l. viciae* secreted protein RapA2. Proteins from the growth-medium supernatants precipitated with trichloro-acetic acid (right panel) and cellular proteins (loading control) from the harvested bacteria (left panel) were separated by SDS-PAGE. The WT was 3841 and strain A1042 is a transductant of 3841 carrying the *praR*::Tn*5* mutation from A963. The culture-supernatant protein arrowed was identified from the culture supernatant of A963 as the product of pRL100451 (RapA2) by MALDI-ToF mass spectrometry of a tryptic digest of the protein eluted from the gel; the statistical score calculated by the mascot program based on five matched fragments was 58 (a score above 50 indicates > 95% confidence). The bacteria were grown for 4 days in Y mannitol medium. The molecular weights of the protein standards in the flanking lanes are indicated.

Several surface components could increase biofilm formation. To test if altered expression of exported surface proteins could be responsible, we mutated *prsD* in the *praR* mutant. The *prsD–prsE* operon encodes components of a Type I secretion system (Finnie *et al*., 1997) required for the secretion of several proteins including potential adhesins and rhizobial adhesion proteins (Raps) (Krehenbrink and Downie, 2008). The *prsD* mutation decreased biofilm formation compared to the *praR* mutant (Fig. 1). Isolation of proteins secreted by the WT, the *praR* mutant and a transductant of WT carrying the *praR* mutation identified a secreted protein present at a higher level in growth-medium supernatant of the *praR* mutants (Fig. 2). Mass spectrometry revealed the protein to be *Rhizobium* adhering protein RapA2, which is secreted via the *prsDE*-encoded Type I secretion system (Krehenbrink and Downie, 2008) and is similar (87% similarity, 83% identity) to the *R. trifolii* adhesin RapA1 that promotes attachment, aggregation and enhanced infection (Mongiardini *et al*., 2008; 2009).

We used microarrays to identify genes upregulated in the *praR* mutant compared with the WT. Thirty-seven genes showed an average induction of twofold or more compared with the WT (Table 1). One gene (RL0149) stood out in that it was strongly induced (over 11-fold) and this gene encodes a predicted transcriptional regulator with homology to PraR (55% similarity; 38% identity using clustalw over a full-length alignment). Most of the other genes apparently upregulated in the *praR* mutant have unknown functions, but three of the genes were of particular interest. One is *praR*, implying that PraR represses its own expression. As expected, one of the genes encodes RapA2, while another encodes RapC, which is related to RapA2. There are three functional *rap* genes in *R.l. viciae* 3841 (Krehenbrink and Downie, 2008) and although *rapB* (RL3911) did not appear in the genes induced more than twofold in the *praR* mutant, it was induced by an average of 1.7-fold (*P* value < 0.015). Therefore the three *rap* genes appear to be among those genes normally repressed by PraR and so it seemed possible that the increased expression of these *rap* genes could cause the enhanced biofilm formation in the *praR* mutant. However we also noted that the two genes, *cadA* (RL2961) and *cadB* (pRL100309) encoding predicted attachment proteins called cadherins (Krehenbrink and Downie, 2008), appeared to be slightly induced in the microarrays (1.8- and 1.4-fold respectively with *P* values of 0.021 and 0.009). Since these proteins are also secreted via the PrsDE system (Krehenbrink and Downie, 2008) and Rap proteins also contain cadherin-like domains (Abdian *et al*., 2013) we considered that *cadA* and *cadB* should be included in our analysis of biofilm formation. Although we focused in this work on genes likely to be repressed directly by PraR, it should be noted that expression of a few genes may be decreased in the *praR* mutant (Table 1); we did not independently confirm decreased expression of these genes, but presumably any decreased expression occurs as an indirect effect of the *praR* mutation.

**Table 1:** Microarray analysis: genes expressed more than or less than twofold in *praR* mutant

Gene ID	Fold up or down	Significance	Gene name	Predicted function of gene product
RL0149	+11.1	1.92E-06		PraR-like transcriptional regulator
RL3192	+3.3	0.017466		Putative ATP-binding component of ABC transporter
RL2937	+3.0	9.63E-06		Putative transmembrane protein
pRL100451	+2.9	5.41E-06	*rapA2*	Putative autoaggregation protein
pRL110037	+2.7	0.004028		Putative two-component fused sensor/regulator
RL4665	+2.7	0.03583		Conserved hypothetical protein
RL4097	+2.7	0.038832		Putative transmembrane cation transporter protein
RL3074	+2.7	0.015478	*rapC*	Putative autoaggregation protein
RL3375	+2.6	0.000292		Conserved hypothetical protein
RL2307	+2.6	0.009037		Conserved hypothetical protein
RL4195	+2.5	0.004917		Putative transmembrane protein
RL1338	+2.5	0.008283	*pmtA*	Putative phosphatidylethanolamine *N*-methyltransferase
RL3959	+2.4	3.71E-05		Conserved hypothetical protein
RL4614	+2.4	0.033157	*rpoH2*	Putative RNA polymerase sigma factor (heat shock)
RL3867	+2.4	0.030485		Conserved hypothetical protein
RL3982	+2.4	0.022785		Conserved hypothetical protein
pRL110129	+2.4	0.021662		Hypothetical protein
RL0390	+2.4	0.035746	*praR*	PraR transcriptional regulator
pRL110586	+2.3	0.029844		Conserved hypothetical protein
RL0610	+2.3	0.013482		Hypothetical exported protein
pRL120342	+2.3	5.75E-05	*hspD*	Putative small heat shock protein
RL3701	+2.3	0.00145		Putative transmembrane protein
RL1165	+2.2	0.02779		Conserved hypothetical protein
RL1513	+2.2	0.000623		Putative FNR/CRP family transcriptional regulator
RL1034	+2.2	0.04639		Conserved hypothetical protein
RL3186	+2.2	0.005128		Putative transmembrane protein
RL3702	+2.2	0.000301		Putative transmembrane protein
RL0506	+2.1	0.004262		Conserved hypothetical protein
pRL110257A	+2.1	0.003572		Conserved hypothetical protein
RL2415	+2.1	0.000762		Hypothetical protein
RL4089	+2.1	6.68E-13	*ibpA*	Putative heat shock protein A hspA
RL3704	+2.1	0.002125	*asfZ*	Putative anti-sigma factor to RL3703
RL4113	+2.1	0.033118		Hypothetical protein
pRL110283	+2.0	0.007827		Putative DNA-binding protein
RL4065	+2.0	0.005843		Conserved hypothetical protein
RL3703	+2.0	0.011679	*ecfZ*	Putative RNA polymerase ECF sigma factor
RL4624	+2.0	4.1E-06	*rpmF*	Putative 50S ribosomal protein L32
pRL110131	−2.0	0.00376		Hypothetical protein
RL4371	−2.1	0.034595		Conserved hypothetical protein
RL2877	−2.1	0.018058		Putative transmembrane transporter
pRL120167	−2.3	0.000351		Putative MFS family transporter
RL2588	−2.3	0.021037	*tyrS1*	Putative tyrosyl-tRNA synthetase
pRL80130	−2.5	0.030906		Putative transmembrane protein
RL1924	−3.2	0.004394		Conserved hypothetical exported protein
RL1925	−3.2	0.018171		Conserved hypothetical protein
RL3670	−5.1	8.47E-05		Conserved hypothetical protein

### Confirmation of PraR-repressed genes using promoter fusions

We made promoter fusion reporter plasmids for the *rapA2, rapB, rapC, praR*, RL0149, *cadA* and *cadB* genes identified from the microarrays as being potentially induced in the *praR* mutant. We also made and used other reporter fusions to test if the expression of other genes was affected by mutation of *praR.* Previously, (Edwards *et al*., 2009), *plyB* was shown to encode a secreted quorum-sensing-regulated glycanase that influences biofilm formation (Russo *et al*., 2006) and so we included reporter fusions with *plyB* and the closely related genes *plyA* and *plyC* (Finnie *et al*., 1998) in our analysis. Since polysaccharides influence biofilms, we also analysed expression of gene fusions with *pssA* and *gmsA* required for the acidic EPS and glucomannan respectively, both of which are important for biofilm formation on root hairs (Williams *et al*., 2008). We also assayed the expression of *rosR* encoding a global regulator of surface rhizobial polysaccharides (Janczarek and Urbanik-Sypniewska, 2013).

**Fig. 3:**
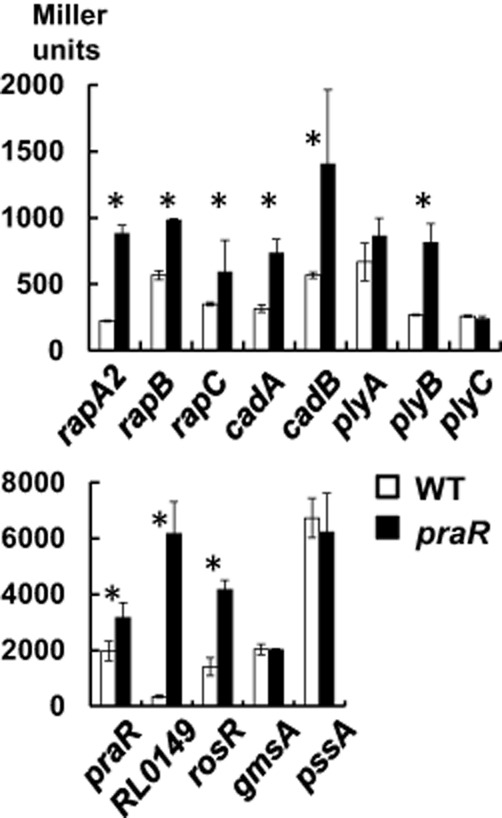
Expression of reporter gene fusions using the promoters of candidate genes potentially repressed by PraR. Levels of expression from the promoters were assayed in either 3841 (WT, open bars) or A963 (*praR*::Tn*5*, black bars) following growth for 48 h in Y mannitol medium. The plasmids containing cloned promoters were pIJ9651 (*rapA2*), pIJ11275 (*rapB*), pIJ11171 (*rapC*), pIJ9686 (*cadA*), pIJ9724 (*cadB*), pIJ11283 (*plyA*), pIJ9252 (*plyB*), pIJ11276 (*plyC*), pIJ11112 (*praR*), pIJ11114 (RL0149), pIJ11196 (*rosR*), pIJ11200 (*gmsA*) and pIJ11195 (*pssA*). Levels of expression of β-glucuronidase (*rapA2* only) or β-galactosidase (all others) are expressed as averages (*n* = 6) ± SD and the asterisks mark those tests in which there is a significant (*P* < 0.05, Student's *t*-test) difference in expression in the *praR* mutant compared with WT. The data are shown in two histograms for simplicity of presentation due to the different levels of expression.

The *rapA2, rapB, rapC, plyB, cadA* and *cadB* genes encoding secreted proteins were induced in the *praR* mutant compared with WT (Fig. 3). The genes encoding the predicted regulators RL0149 and RosR were also increased in expression in the *praR* mutant. We also confirmed that PraR represses its own expression (Fig. 3). In contrast, mutation of *praR* did not significantly induce *gmsA* (required for glucomannan synthesis), or *pssA, plyA* and *plyC* (associated with the synthesis and cleavage of the acidic exopolysaccharide) (Fig. 3).

### Identification of PraR-binding promoters

**Fig. 4:**
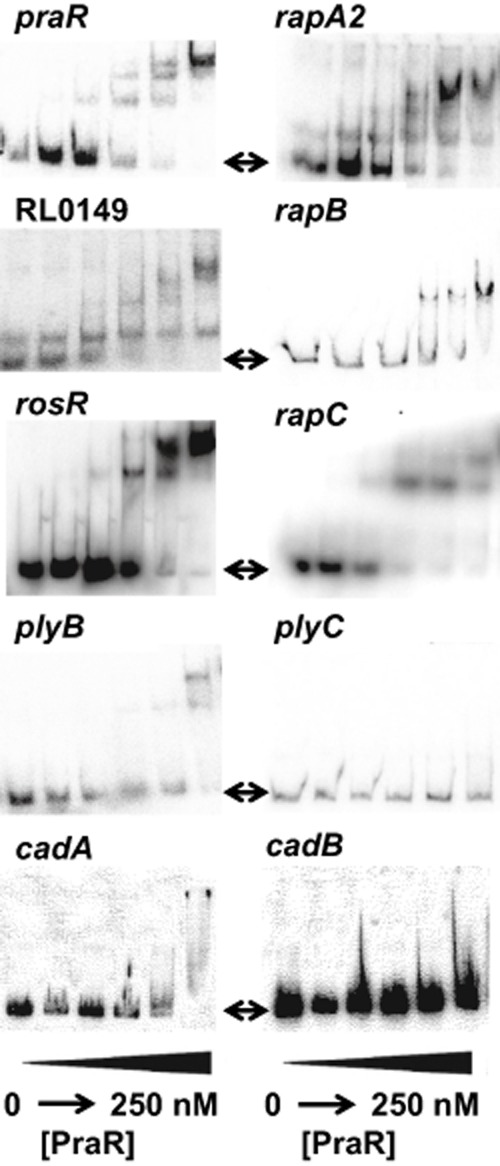
*In vitro* analysis of PraR binding to different promoters. Radioactively labelled promoters of the genes indicated were incubated with a purified PraR maltose-binding protein fusion (from left to right on each gel: 0, 16 nM, 32 nM, 64 nM, 125 nM and 250 nM protein). After the reactions the samples were separated by non-denaturing gel electrophoresis and the radioactively labelled bands were imaged using a phosphorimager as described previously (Frederix *et al*., 2011). The arrows indicate the sizes of unshifted fragments.

The enhanced expression of *rapA2, rapB, rapC, cadA, cadB, plyB, rosR, praR* and *RL0149* in the *praR* mutant could be direct, due to release of repression by PraR, or indirect, e.g. due to the altered expression of a transcriptional regulator. At about 30–62 nM, PraR bound to the promoters of *rapA2, rapB, rapC, rosR, praR* and RL0149 (Fig. 4), reducing the mobility of radioactively labelled promoter fragments during electrophoresis as seen previously with the *rhiR* promoter (Frederix *et al*., 2011). This, together with the complete lack of binding of PraR to the *plyC* and *cadB* promoters (Fig. 4) demonstrates that PraR specifically interacts with the *rapA2, rapB, rapC, rosR, praR* and RL0149 promoters and so most probably directly represses their expression. There may be weaker binding of PraR to the *cadA* and *plyB* promoters, but this required higher levels of PraR (62–250 nM). We used MEME (Bailey and Elkan, 1994) to analyse the promoter fragments of *rhiR*, *rapA2, rapB, rapC, rosR, praR* and *RL0149* for a consensus PraR binding site that was also present in the PraR-binding 20-nucleotide fragment identified previously (Frederix *et al*., 2011). This identified the consensus motif TTGCAA (Fig. S1). This conserved sequence was not present in the *cadA, cadB, plyB* or *plyC* promoters.

Since mutation of *praR* increased expression of most of the PraR-repressed genes only two- to threefold, PraR may repress expression of other genes not included in Table 1 because their expression increased less than twofold. As expected from the reporter fusion, PraR did not bind to the *plyC* promoter, but unexpectedly, PraR was not observed to bind to the *cadB* promoter (Fig. 4).

Previously (Frederix *et al*., 2011) we observed that the antirepressor CinS interacts with PraR, preventing PraR binding to the *rhiR* and *raiR* promoters. Since PraR and CinS can interact in the absence of cognate promoters, we expected that CinS would antagonize PraR binding to other promoters. We tested this using gel shift assays with the *praR* and *rapA2* promoters and, as expected, CinS blocked PraR binding (Fig. S2).

Given the similarity between PraR and RL0149, we thought that there might be cross-regulation involving these two proteins. We made a mutation in the RL0149 gene and tested the expression of *praR*, *rhiR, rosR* and *rapA2* in the mutant and saw no significant changes in gene expression or biofilm formation (data not shown). We also made a *praR*-RL0149 double mutant and saw no difference in the expression of *praR* or RL0149. Therefore the role of the RL0149 gene remains enigmatic.

### A light emission (*lux*) assay shows that enhanced root attachment by the *praR* mutant requires Rap proteins and glucomannan

**Fig. 5:**
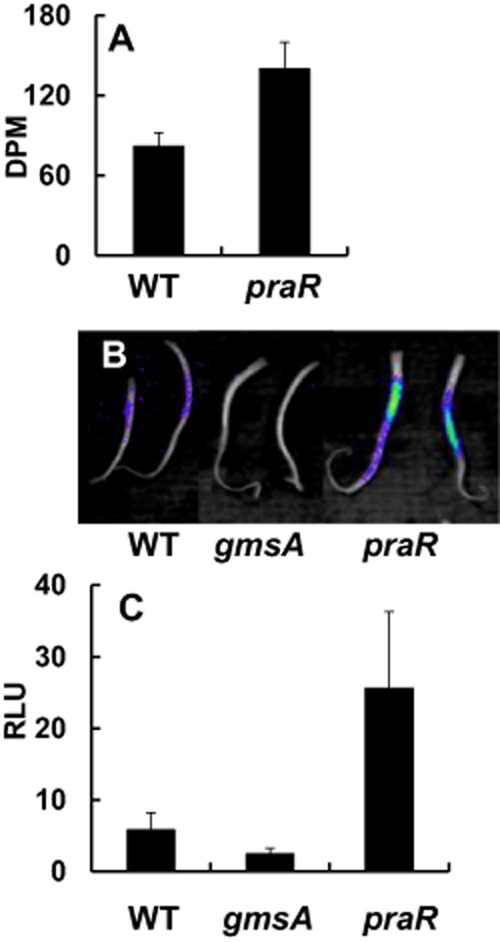
Enhanced root attachment by the *praR* mutant shown by radiolabelling and luminescence.A. *R.l. viciae* strains 3841 (WT) and A963 (*praR*::Tn*5*) were labelled with ^3^H-leucine and incubated with roots excised from pea seedlings. The roots were washed three times and then the bound radioactivity on each root was measured using a scintillation counter. The data show average counts determined using 10 roots (± SD).B. A similar attachment assay as in A was performed using strains 3841 (WT), A963 (*praR*) and A1042 (*gmsA*); instead of labelling with ^3^H-leucine, each strain carried the *lux* plasmid pIJ11282 expressing the *luxCDABE* operon (Fig. S1) and bacterial attachment was photographed using a NightOWL LB983 ultrasensitive light-imaging system. Strain A1042 (*gmsA*) was included to confirm that this method can document the observed decrease in attachment of the *gmsA* mutant (Williams *et al*., 2008).C. Attachment of 3841 (WT), A963 (*praR*) and A1042 (*gmsA*) all carrying pIJ11282 was done as in B, but quantified using a luminometer. Data shown are averages of Relative Light Units (RLU) based on separate measurements with 10 roots (± SD) and all are significantly different from each other (*P* = < 0.05, Student's *t*-test).

To examine whether the altered biofilm formation of the *praR* mutant was correlated with changes in root attachment, cells of the *praR* mutant and WT were labelled with ^3^H-leucine and then incubated with pea root segments; after several washes, attachment was then measured by determining the levels of radioactivity attached to the roots. This revealed that more of the *praR* mutant attached than the WT (Fig. 5A).

This radioactivity-based assay for attachment was inconvenient and so we devised an alternative strategy to measure attachment. We made a stably inherited plasmid (pIJ11282) constitutively expressing the *lux* operon (Fig. S3), transferred it into *R.l. viciae* 3841, and inoculated the resulting strain onto the roots of pea seedlings; light emission was then measured after washing off loosely attached bacteria, revealing that the bacteria were mostly attached to the region of the root above the tip, where young root-hairs are growing (Fig. 5B). In previous work (Williams *et al*., 2008), attachment of *R.l. viciae* to vetch root hairs was shown to require the surface glucomannan polysaccharide, which binds to a plant lectin expressed on root hairs (Laus *et al*., 2006). Blocking the production of the glucomannan by mutating the glucomannan synthase gene *gmsA* reduced attachment to pea roots measured using this *lux*-dependent light emission assay (Fig. 5B). In contrast, the *praR* mutant inoculated onto pea roots showed higher levels of light emission than WT (Fig. 5B); quantification using a luminometer confirmed that the *praR* mutant attached better than the WT (Fig. 5C). Colony counts of WT and *praR* mutant bacteria attached to pea roots also demonstrated that the *praR* mutant attaches at two- to threefold higher levels than WT, with the *praR* mutant attaching at 7.8 ± 0.19 × 10^5^ cfu compared with the WT at 3.15 ± 0.07 × 10^5^ cfu. Since the results with the *lux*-marked bacteria matched the enhanced attachment assayed by the radioactive assay, and by bacterial counts, we used the *lux*-based assay for further experiments.

**Fig. 6:**
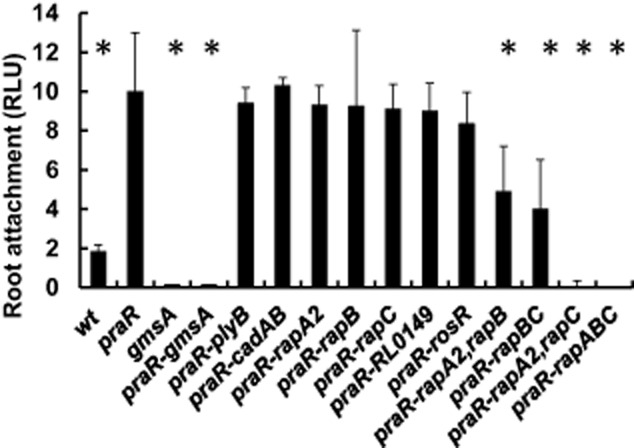
Enhanced root attachment by the *praR* mutant requires *gmsA* and *rap* genes. Attachment of the *R.l. viciae* strains was assayed using bacteria expressing the constitutively expressed *lux* operon on pIJ11282 following incubation of the bacteria with excised roots from pea seedlings and subsequent washing of the roots. The strains used were 300 (WT), A1345 (*praR*), A1208 (*gmsA*), A1367 (*praR*, *gmsA*), A1372 (*praR*, *plyB*), A1383 (*praR*, *cadA*, *cadB*), A1328 (*praR*, *rapA2*), A1416 (*praR*, *rapB*), A1374 (*praR*, *rapC*), A1385 (*praR*, *rosR*), A1427 (*praR*, *rapA2*, *rapB*), A1428 (*praR*, *rapB*, *rapC*), A1430 (*praR*, *rapA2*, *rapC*) and A1429 (*praR*, *rapA2*, *rapB*, *rapC*). Light emission of the attached bacteria was quantified using a luminometer. Data shown are averages of Relative Light Units (RLU) based on separate measurements with 10 roots (± SD) for each strain; averages significantly different (Student's *t*-test *P* = < 0.05) from the *praR* mutant are marked (*).

We made a *praR-gmsA* double mutant to test whether increased expression of genes normally repressed by PraR could restore root attachment by the *gmsA* mutant. The *praR-gmsA* double mutant was reduced in attachment compared with the *praR* mutant (Fig. 6); this would fit with a model in which genes induced by mutation of *praR* enhance attachment following an initial lectin-mediated binding to the GmsA-determined glucomannan.

Since PraR represses expression of *rapA2, rapB, rapC, cadA, cadB, plyB, rosR* and R0149, we introduced mutations in each of these genes into the *praR* mutant to determine which gene(s) were required for the enhanced root attachment by the *praR* mutant. None of these mutations significantly decreased the enhanced attachment of the *praR* mutant (Fig. 6). Since the Rap proteins are structurally similar, we made all combinations of the *rap* gene mutations in the *praR* mutant and tested their attachment. Likewise since CadA and CadB are related we made a double *cadA, cadB* mutant. The combination of mutations in both *rapA2* and *rapC* abolished the enhanced attachment of the *praR* mutant whereas the combination of mutations in *rapA2* and *rapB*, or *rapC* and *rapB* decreased attachment (Fig. 6). Therefore we conclude that the enhanced attachment in the *praR* mutant is primarily due to increased levels of the secreted adhesins RapA2 and RapC, although RapB also plays a role. In contrast, the *cadA, cadB* double mutant was unaffected for attachment (Fig. 6).

### The *praR* mutant out-competes the WT for nodule infection and this requires both the glucomannan and the Rap proteins

**Fig. 7:**
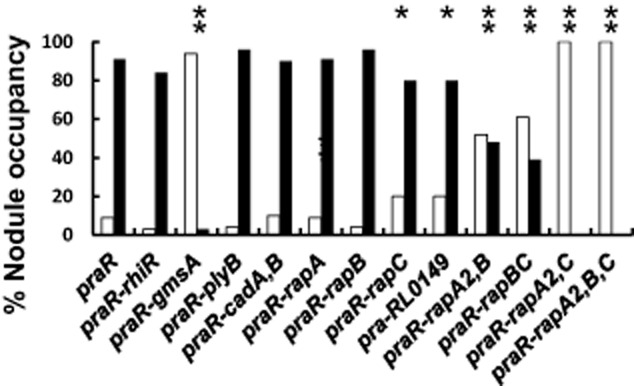
The *R.l. viciae* *praR* mutant has increased competitiveness for nodule infection and this requires the *rap* and *gmsA* genes. Strains A1345 (*praR*), A1208 (*gmsA*), A1367 (*praR*, *gmsA*), A1372 (*praR*, *plyB*), A1149 (*praR*, *rhiR*), A1383 (*praR*, *cadA*, *cadB*), A1328 (*praR*, *rapA2*), A1416 (*praR*, *rapB*), A1374 (*praR*, *rapC*), A1427 (*praR*, *rapA2*, *rapB*), A1428 (*praR*, *rapB*, *rapC*), A1430 (*praR*, *rapA2*, *rapC*) and A1429 (*praR*, *rapA2*, *rapB*, *rapC*) were each mixed in a 1:1 ratio with 3841 (WT) and inoculated onto peas. Bacteria were isolated from surface-sterilized individual nodules and the released bacteria were identified based on their antibiotic resistance. The graph shows the % of WT (open bars) or mutants (black bars) recovered from nodules; those results that were significantly different (chi-squared test) from A1345 (*praR*) + 3841 (WT) are marked (**P < 0.01; *P < 0.05). Each analysis is based on identification of mutant or WT bacteria isolated from at least 100 nodules from at least five separate plants and excludes potentially mixed infections.

Root hair attachment can be related to competitiveness for nodule infection (Williams *et al*., 2008), and so we tested whether the *praR* mutant had a competitive advantage when co-inoculated with WT. To assay this, the *praR::*Tn*5* mutation was transduced into strain 300, the streptomycin-sensitive parent of *R.l.* bv. *viciae* 3841, forming A1132. We first ascertained that strain 300 was equally competitive with strain 3841: when inoculated onto pea roots at a 1:1 ratio, the numbers of nodules formed by each strain was not significantly different from predicted (chi-squared test). Strain A1132 (*praR*), which grew with a similar doubling time as WT, was assayed for competitiveness in infection of pea nodules by co-inoculating A1132 with an equivalent amount of strain 3841 (WT) and then scoring bacteria from individual nodules for resistance to kanamycin (A1132) or streptomycin (3841). The *praR* mutant outcompeted the WT (Fig. 7) indicating that the increased attachment results in increased infection.

To determine if the increased infection by the *praR* mutant required the glucomannan previously shown to be required for infection competitiveness (Williams *et al*., 2008), we tested the ability of the *praR-gmsA* double mutant to compete with WT. Since this mutant was a poor competitor (Fig. 7), we can conclude that the increased expression of *rap* genes cannot compensate for the loss of glucomannan in infection. The *rapA* or *rapB* mutations had no significant effect on the infection competitiveness of the *praR* mutant, whereas the *rapC* mutation slightly decreased its competitiveness (Fig. 7). The combination of mutations in *rapA2* and *rapB*, or *rapC* and *rapB* further decreased competitiveness, while strains carrying combination of mutations in both *rapA2* and *rapC* abolished the enhanced competitiveness of the *praR* mutant (Fig. 7). As with all the mutants tested (Fig. 7), the *praR* mutant carrying mutations in all three *rap* genes grew with the same doubling time as WT in planktonic culture in mannitol minimal medium and produced normal numbers of pink nodules when inoculated alone, showing that the effects of the mutations are primarily on the competitiveness, rather than effects on growth or nodule infection *per se*.

In contrast to these additive effects of *rap* mutations on competitiveness, the combination of the *cadA* and *cadB* mutations did not decrease nodulation competitiveness of the *praR* mutant. Likewise, introduction of a *rhiR* mutation into the *praR* mutant did not affect its increased nodule infection, demonstrating that the quorum-sensing genes induced by RhiR are not required for the increased infection potential (Fig. 7). Mutation of RL0149 slightly decreased competitiveness (Fig. 7), indicating that this gene may play a subtle role in competitive nodule infection.

It is evident that those mutations that decreased root attachment by the *praR* mutant also decreased its ability to outcompete the WT for nodule infection. The enhanced attachment is not mediated via the RhiR regulon but requires the Rap proteins. Our observations are compatible with a model in which glucomannan-mediated attachment occurs first and that subsequent Rap-mediated adhesion (either between bacteria or enhancing bacterial attachment to roots) increases the infection potential of a rhizobial strain.

Although the *rap* genes are clearly required for competitive nodule infection and root attachment in a *praR* mutant, there is a possibility that this requirement is only seen in the *praR* mutant background. As proof of principle of the role of Rap proteins in competitive nodule infection we made a derivative of WT (3841) carrying mutations in *rapA2* and *rapC*. This mutant (A1480) nodulated peas normally, but was completely defective for competitive nodule infection: when mixed 1:1 with strain 3841 all 192 nodules tested were occupied by 3841. There was also a complete loss of root attachment (0.05 ± 0.005 RLU) equivalent to that shown for the *praR, rapA2, rapC* mutant in Fig. 6. Therefore we conclude that the Rap proteins are essential for normal attachment and competitive nodule infection in the presence or absence of PraR.

### The *rapA2*, *rapB* and *rapC* genes are differentially regulated

**Fig. 8:**
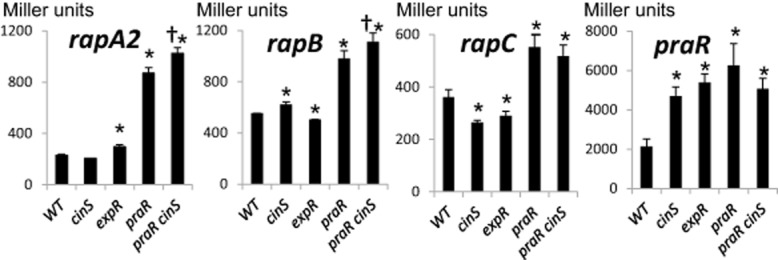
Differential regulation of *rapA2*, *rapB*, *rapC* and *praR* in *cinS*, *expR* and *praR* mutants. Levels of expression from the promoters were assayed in either 3841 (WT), A1229 (*cinS*), A1216 (*expR*), A963 (*praR*) or A1312 (*praR*, *cinS*) following growth for 48 h in Y mannitol medium. The plasmids containing cloned promoters were pIJ9651 (*rapA2*), pIJ11275 (*rapB*), pIJ11171 (*rapC*) or pIJ11112 (*praR*). Levels of expression of β-glucuronidase (*rapA2* only) or β-galactosidase (all others) are expressed as averages (*n* = 6) ± SD in Miller units. Those tests in which there is a significant (*P* < 0.05, Student's *t*-test) difference in expression are marked: the asterisk (*) indicates significant differences compared with WT and the dagger (†) indicates significant differences in expression in A1312 (*praR*, *cinS*) compared with expression in A963 (*praR*). The reduced expression of *rapA2* in the *cinS* mutant was just outside the range of significance (*P* = 0.0504).

**Fig. 9:**
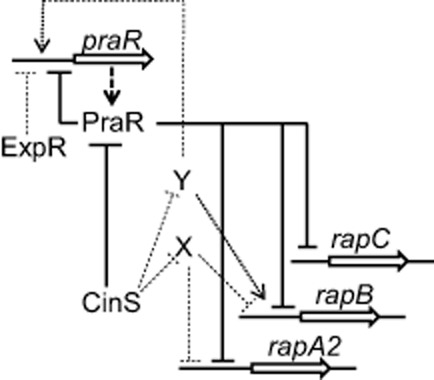
Model of regulation by PraR. Expression from the *praR* promoter is directly repressed by PraR (autoregulated) and since mutation of *expR* increased *praR* expression we speculate that ExpR also represses *praR*. PraR directly represses expression of *rapA2*, *rapB*, *rapC* (shown), *rosR* and RL0149 (not shown). CinS, which is expressed in a population-density-dependent manner via the CinR–CinI-regulated quorum-sensing system, binds to PraR attenuating its repression. Since expression of *rapA2* and *rapB* increased in the *cinS*, *praR* mutant compared with the *praR* mutant (Fig. 8), CinS must interact with another protein or proteins. The increased *rapA2* and *rapB* expression could be due to CinS binding to and inactivating an unknown repressor (X). The increased expression of both *praR* and *rapB* in the *cinS* mutant (Fig. 8) could be due to CinS attenuating the activity of an unknown positive transcriptional regulator (Y) as indicated; alternatively increased levels of repressor X in the *cinS* mutant could repress expression of another repressor of *praR* (not shown). Full arrows and lines are based on tested interactions; dotted arrows and lines are speculations that fit the gene expression patterns.

One apparent anomaly is that in addition to the *praR* mutation increasing biofilms, mutation of *cinS* also increased biofilms (Edwards *et al*., 2009; Frederix *et al*., 2011). Since CinS reduces PraR-mediated repression, mutation of *cinS* would be expected to enhance PraR-mediated repression and therefore decrease (rather than increase) biofilm attachment by reducing *rap* gene expression. Therefore we measured *rapA2*, *rapB* and *rapC* expression in a *cinS* mutant; as anticipated, there was a small decrease in *rapC* expression and a small decrease in *rapA2* expression that is probably real but was just outside the significance range. Surprisingly there was a small increase in *rapB* expression (Fig. 8) possibly explaining the enhanced biofilm in the *cinS* mutant. Another potential regulator is ExpR, because mutation of *expR* also increased biofilm formation (Edwards *et al*., 2009); this might be due to by the increased *rapA2* expression in the *expR* mutant (Fig. 8). One way in which differential regulation of the *rap* genes could occur could be for CinS to influence *rap* gene expression by interacting with another regulator (Fig. 9). To test this possibility, we introduced the *cinS* mutation into the *praR* mutant; the resulting increased *rapA2* and *rapB* expression in the *cinS, praR* double mutant (Fig. 8) shows (as indicated in Fig. 9) that CinS must affect some regulator in addition to PraR (attenuating repression by another protein being the simplest explanation). Regulation of *praR* itself is complex, because mutation of *cinS, praR* or *expR* increased *praR* expression (Fig. 8). A working model for regulation of PraR expression and activity is shown in Fig. 9.

## Discussion

The optimal mode of growth for many bacteria in nature is in biofilms, and this is particularly true for soil bacteria that can attach to the roots and root hairs of growing plants, which secrete nutrients into the rhizosphere. The switch between planktonic and biofilm growth is an important lifestyle switch and is coupled to changes in expression of many genes. There are two very good reasons why rhizobia attach to root hairs: one is to benefit from secreted nutrients and the other is to attach specifically to host legume root hairs so that by being well positioned they have a better chance of infecting roots and growing exponentially during infection and nodule development (Downie, 2010).

In *R.l. viciae*, we have shown that PraR is a regulator that modulates gene expression during adaptation to biofilm growth. PraR represses the expression of several genes including those encoding three rhizobial adhering proteins (RapA, RapB and RapC), a predicted cadherin (CadA), an EPS glycanase (PlyB) and at least two regulators, one of which (RL0149) is similar to PraR and the other of which (RosR) is a global regulator of production of surface polysaccharides (Janczarek and Urbanik-Sypniewska, 2013). Several other genes may be repressed by PraR, but since mutating *praR* causes only a small (two- to threefold) induction of those genes identified as direct targets, microarrays are not the best way to identify genes regulated by PraR; chromatin immunoprecipitation would identify a greater range of PraR regulated genes. Although the increase in expression of the *rap* genes in the *praR* mutant is relatively low, it is sufficient to initiate changes in attachment to roots, leading to increased nodulation competitiveness in mixed inoculations. Possibly other PraR-regulated genes including *plyB, rosR* and the cadherins may play a role in attachment/biofilm formation that would only be revealed under different test conditions. The fact that *praR* remains functional in *R.l. viciae* must mean that there is another stage in the lifestyle where it is important that the PraR-regulated genes are repressed. This could e.g. be explained by the requirement for rhizobia to move around in the rhizosphere and a lifestyle with too much attachment could hamper other aspects of their competitiveness in the wild.

PraR and its orthologue PhrR are well conserved within the α-proteobacteria (Akiba *et al*., 2010), but their regulation and the genes they regulate seem to be rather different, even among rhizobia. Low pH induced *phrR* in *S. medicae*, but *praR* appears not to be induced by low pH in *R.l. viciae* or *A. caulinodans* (Akiba *et al*., 2010; Frederix *et al*., 2011). PraR-regulated genes in *A. caulinodans* were identified using microarrays (Akiba *et al*., 2010), but none of the top 10 genes induced in the *A. caulinodans praR* mutant was induced by mutation of *praR* in *R.l. viciae*. PraR in *A. caulinodans* represses seven *reb* genes that are thought to determine the production of inclusion bodies; it is the enhanced expression of these *reb* genes that causes the Fix^-^ phenotype of the *A. caulinodans praR* mutant (Akiba *et al*., 2010). In *R.l. viciae* there are no *reb*-gene homologues and the *praR* mutation does not cause a Fix^-^ phenotype. One of the other genes (AZC-1189) that was most upregulated by mutation of *praR* in *A. caulinodans* also has no homologue in *R.l. viciae* and the homologues of the two induced flagellar genes (AZC-3379 and AZC-2699) did not appear to be enhanced in expression in the *R.l. viciae praR* mutant.

As illustrated (Fig. 9), the control of PraR activity in *R.l. viciae* is affected by quorum-sensing induction of CinS, which binds to PraR (Frederix *et al*., 2011), decreasing PraR-mediated repression; in this way PraR-repressed genes can be induced as the population density increases. However the regulation is complex because PraR represses its own expression (Fig. 9). There are parallels with the regulation of biofilm formation by SinR in *B. subtilis*; SinR represses expression of exopolysaccharide genes and a gene encoding a secreted protein that is required for normal biofilm formation (Kearns *et al*., 2005; Chu *et al*., 2006). Repression by SinR is relieved by SinI which binds to SinR, preventing SinR from acting as a repressor. CinS is not structurally similar to SinI, but by binding to PraR it can affect EPS production by increasing RosR expression and can allow increased expression of the three Rap proteins. As in *B. subtilis*, the regulation of *R.l. viciae* biofilm genes is complex, because another regulator, ExpR represses *praR* expression. Furthermore, mutations in *expR* and *cinS* have different effects on the regulation of the different *rap* genes (Fig. 9). It seems likely that differential expression of the *rap* genes can allow bacteria to attach at different stages of growth. For example *rapB* expression increased in the absence of CinS, a situation that would prevail at low population densities, whereas the expression of *rapC* is higher as CinS levels increase as seen at high population densities. However these interpretations are based on averages of gene expression across a population and this is unlikely to hold in biofilms. For example in *B. subtilis* biofilms, *sinI* was only expressed in a subpopulation of the cells whereas *sinR* was expressed in all cells (Chai *et al*., 2008).

The enhanced root attachment and increased infection competitiveness of the *praR* mutant appear to be due primarily to the increased expression of the *rap* genes (Fig. 9) and this is consistent with what was seen with increased expression of *rapA1* on a plasmid in *R.l. trifolii* (Mongiardini *et al*., 2008). The enhanced root-hair attachment and infection of root hairs by the *praR* mutant was abolished by the *gmsA* mutation that prevents the formation of glucomannan. This polarly expressed polysaccharide is specifically recognized by a legume-specific root-hair-tip localized plant lectin and has been shown to be important for the initial step of rhizobial binding (Laus *et al*., 2006). In the absence of the glucomannan, infection can occur normally, but the bacteria are uncompetitive (Williams *et al*., 2008). In addition, the acidic EPS plays an essential role in root-hair infection. Since the Rap proteins bind to this EPS and are required for enhanced attachment to roots, we propose that after glucomannan-plant-lectin mediated attachment, Rap-mediated interactions between bacteria will permit an accumulation of bacteria on root hairs and that this promotes competitiveness. Bacterially made extracellular cellulose also promoted accumulation of on root hairs, but this cellulose-mediated effect did not significantly contribute to competitiveness (Williams *et al*., 2008). In fact cellulose production by *R.l. viciae* even appeared to inhibit initiation of infection in some circumstances (Laus *et al*., 2005). Based on these data, we propose that the glucomannan-plant lectin binding followed by the Rap-EPS interaction play key roles in competitive infection whereas the cellulose mediated stabilization of biofilms is important for bacterial growth on root-hairs.

## Experimental procedures

### Bacterial growth, assays of β-galactosidase and β-glucuronidase and competitive nodule infection

*Rhizobium leguminosarum* strains were grown at 28°C in TY medium (Beringer, 1974), AMS (minimal) medium (Poole *et al*., 1994) containing 10 mM NH_4_Cl and 30 mM glucose, or Y minimal medium (Sherwood, 1970) buffered with MOPS (Williams *et al*., 2008) and containing mannitol (0.2% w/v). *E. coli* was grown at 37°C in L medium (Sambrook *et al*., 1989). Antibiotics were added as appropriate to maintain selection for plasmids. β-Galactosidase was assayed as Miller units as described previously (Edwards *et al*., 2009) using at least three independent cultures. β-Glucuronidase was assayed using the same method except that the β-galactosidase substrate *o*-nitrophenol β-d-galactopyranoside was replaced with *p*-nitrophenol β-d-glucopyranoside. Conjugal matings using a helper plasmid and transductions using RL38 phage were done as described (Buchanan-Wollaston, 1979; Figurski and Helinski, 1979). DNA cloning, ligations and transformations were done using standard methods (Sambrook *et al*., 1989). Biofilm formation in flasks and microtitre plates was measured as described previously (Edwards *et al*., 2009). Competitive nodule infection experiments were carried out as described previously (Williams *et al*., 2008) using at least 100 nodules from a minimum of five separate plants in each test. Some crushed nodules (usually around 5% and always less than 20%) apparently contained two bacterial genotypes and these were excluded from the analyses; this co-infection of pea nodules may occur due to two infection threads infecting one nodule or the fusion of two nodules into what appears to be a single mutilobate nodule.

### Construction of strains and plasmids

Strains, plasmids and primers used in this study are listed in Tables 2 and 3 and Tables S1 and S2. *R. leguminosarum* 3841 mutants with Tn*5* insertions in *cadA* (RL2961)*, cadB* (pRL100309), *RL0149, rosR* (RL1379), *plyB* (RL3023), or *rapB* (RL3911), were identified in pools of mutants from a Tn5 library using gene-specific and Tn5-specific PCR primers (Table S2) as previously described (Williams *et al*., 2008). To make A1206 (*rapA2Ω*Spec^R^)*, rapA2* (pRL100451) with 1 kb flanking regions was amplified by PCR using primers *rapA2*F and *rapA2*R (Table S2) and the product was digested with KpnI and SpeI and cloned into pJQ200KS; the spectinomycin resistance gene on pMP45*Ω* was then amplified (primers specF and specR) and cloned as an AccIII fragment into the AccIII site in *rapA2* in pJQ200KS. The *rapA2Ω*spec^R^ allele was then recombined into strain 3841 selecting for spectinomycin and sucrose-resistant transconjugants (Quandt and Hynes, 1993) and the *rapA2Ω*specR allele in A1206 was confirmed by PCR. To make the *rapC* mutant A1362, an internal (347 bp) fragment of the *rapC* gene (RL3074) was amplified using PCR (primers *rapC*F and *rapC*R, Table S2) and cloned into pK19mob using the XhoI and HindIII restriction sites introduced on the primers. The resulting plasmid was conjugated into strain 3841 selecting on neomycin (400 μg ml^−1^) and a single-crossover integration into *rapC* was verified by PCR.

**Table 2:** Bacterial strains

Strain	Description	Source
300	Wild-type *R. leguminosarum*	Johnston and Beringer (1975)
3841	*R. leguminosarum* 300; strep^R^	Johnston and Beringer (1975)
A898	3841 *prsD::*Tn*5*ΩGent	Krehenbrink and Downie (2008)
A920	3841 *rhiR::*Tn*5*	This work
A963	3841 *praR::*Tn*5*	Frederix *et al*. (2011)
A1024	3841 *rhiR::*Tn*5*ΩGent	This work
A1042	3841 *praR::*Tn*5* transduced from A963	This work
A1045	3841 *gmsA::*Tn*5*ΩGent	Williams *et al*. (2008)
A1132	300 *praR::*Tn*5* transduced from A963	This work
A1149	3841 *praR::*Tn*5, rhiR::*Tn5ΩGent	This work
A1161	3841 *praR::*Tn*5, prsD::*Tn*5*ΩGent	This work
A1167	3841 *praR::* Tn*5*ΩSpec	This work
A1176	3841 *rosR::*Tn*5*	This work
A1206	3841 *rapA2*ΩSpec^R^	This work
A1208	300 *gmsA::*Tn*5*ΩGent	Williams *et al*. (2008)
A1216	3841 *expR*ΩpK19mob	Frederix *et al*. (2011)
A1229	3841 *cinS*ΩApra	Frederix *et al*. (2011)
A1253	3841 *cadB::*Tn*5*	This work
A1254	3841 *cadA::*Tn*5*	This work
A1261	300 *cadB::*Tn*5* transduced from A1253	This work
A1263	3841 *cadB::*Tn*5*ΩGent	This work
A1312	3841 *praR::*Tn*5*ΩSpec *cinS*ΩApra	Frederix *et al*. (2011)
A1328	300 *praR::*Tn*5* with *rapA2*ΩSpec^R^ transduced from A1206	This work
A1327	3841 *RL0149::*Tn*5*	This work
A1340	300 *RL0149::*Tn*5* transduced from A1327	This work
A1345	300 *praR::*Tn*5*ΩSpec	This work
A1362	3841*rapC*Ωpk19mob	This work
A1363	300 *praR::*Tn*5*ΩSpec *cadA::*Tn*5*	This work
A1365	3841 *plyB::*Tn*5*	This work
A1367	300 *praR::*Tn*5*ΩSpec *gmsA::*Tn*5*	This work
A1368	300 *praR::*Tn*5*ΩSpec *RL0149::*Tn*5*	This work
A1370	300 *praR::*Tn*5*ΩSpec *rhiR::*Tn*5*	This work
A1372	300 *praR::*Tn*5*ΩSpec *plyB::*Tn*5*	This work
A1374	300 *praR::*Tn*5*ΩSpec *rapC*Ωpk19mob	This work
A1376	3841 *rapB*::Tn*5*	This work
A1383	300 *praR::*Tn*5*ΩSpec *cadA::*Tn*5 cadB::*Tn*5*ΩGent	This work
A1384	300 *praR::*Tn*5*ΩSpec *prsD::*Tn*5*	This work
A1385	300 *praR::*Tn*5*ΩSpec *rosR::*Tn*5*	This work
A1416	300 *praR::*Tn*5*ΩSpec *rapB::*Tn*5*	This work
A1417	300 *rapB::*Tn*5* transduced from A1376	This work
A1425	3841 *rapB::*Tn*5*ΩGen	This work
A1426	3841 *rapC::*Tn*5*ΩApra	This work
A1427	300 *praR::*Tn*5 rapA2*ΩSpec *rapB::*Tn*5*ΩGent	This work
A1428	300 *praR::*Tn*5*ΩSpec, *rapB::*Tn*5*ΩGent, *rapC*Ωpk19mob	This work
A1429	300 *praR::*Tn*5 rapA2*ΩSpec *rapB::*Tn*5*ΩGen *rapC::*Tn*5*ΩApra	This work
A1430	300 *praR::*Tn*5 rapA2*ΩSpec *rapC::*Tn*5*ΩApra	This work
A1480	300 *rapA2*ΩSpec *rapC::*Tn*5*ΩApra	This work

**Table 3:** Plasmids

Plasmid	Description	Reference
pIJ9252	*plyB′-lacZ*	Edwards *et al*. (2009)
pIJ9651	*rapA2′-gus*	This work
pIJ9686	*cadA′-lacZ*	This work
pIJ9724	*cadB′-lacZ*	This work
pIJ11112	*praR′ -lacZ*	This work
pIJ11114	*RL0149′-lacZ*	This work
pIJ11171	*rapC′-lacZ*	This work
pIJ11195	*pssA′-lacZ*	This work
pIJ11196	*rosR′-lacZ*	This work
pIJ11200	*gmsA′-lacZ*	This work
pIJ11275	*rapB′-lacZ*	This work
pIJ11276	*plyC′-lacZ*	This work
pIJ11283	*plyA′-lacZ*	This work
pIJ11268	Broad host range; promoterless *luxCDABE* Tet^R^, Amp^R^	This work
pIJ11282	Derivative of pIJ11268 with *nptII* promoter	This work
pIJ11304	For exchanging Kan^R^ in Tn*5* to Apra^R^	This work

Mutations in strain 300 were transduced from the appropriate 3841 strains containing single mutations. To facilitate construction of strains carrying multiple mutations, the *nptII* gene on Tn*5* in some mutants was replaced by the spectinomycin or gentamicin resistance genes on plasmids pJQ173 and pJQ175 respectively as described previously (Quandt *et al*., 2004). Also, an apramycin-resistance plasmid was made by amplifying the apramycin resistance gene on pIJ733 using the primers pApra and *aac*5 (Table S2) and cloning it as a BamHI fragment into BamHI-cut pJQ173 to give plasmid pIJ11304 which was used in the same way as pJQ173. The *praR::*Tn5 mutation was transduced into 3841 (WT) to make A1042; to make *praR* mutants with mutations in additional genes, A1042 (*praR::*Tn5) or A1345 (*praR*::Tn*5*ΩSpec) were transduced using phage RL39 propagated on strains carrying the appropriate single mutations, selecting with appropriate antibiotics. One of these mutants, A1363 (*praR, cadA*) was then transduced with phage from A1261 to make the *praR, cadA, cadB* mutant A1383. A1374 (*praR, rapC*) was transduced with phage from A1425 to make the *praR, rapB, rapC* mutant A1428. The *rapA2, praR* double mutant A1328 was made by transduction of A1132 (*praR*::Tn*5*) using phage from A1206. The *rapB*::Tn*5*ΩGent and *rapC*Tn*5*ΩApra alleles were introduced into A1328 to make A1427 and A130 respectively, by transduction using phage propagated on A1425 or A1426. The *praR, rapA2, rapB, rapC* mutant A1429 was generated by transduction of A1427 with phage from A1426. A1480 a derivative of 300 carrying mutations in *rapA2* and *rapC* was made by sequential transduction with phage from A1206 and A1426.

Promoter fusion reporter plasmids were constructed by cloning PCR-amplified promoters into the *lac*Z reporter plasmid pMP220 (Spaink *et al*., 1987) or the *gus* reporter plasmid pRU1156 (Karunakaran *et al*., 2005). Promoter amplification was carried out using the gene-specific primers listed in Table S2. Either restriction sites (underlined) were introduced during PCR or the amplification product was first cloned into pGEM T-easy and then excised as an EcoRI fragment. The correct orientation of the insert was determined by PCR using a vector and gene-specific primers.

### Protein work

Protein electrophoresis and MALDI-ToF were done as described (Krehenbrink and Downie, 2008). Gel shift assays were done as described previously (Frederix *et al*., 2011) with the same promoter fragments amplified to make the reporter-plasmid fusions described above. These fragments were amplified using the primers listed in Table S2 and then end-labelled using γ^32^P-ATP. Each 20 microlitres of reaction buffer (20 mM TrisHCL, 100 mM NaCl, 5 mM MgCl_2_, 1 mM EDTA, 0.5 mM dithiothreitol and 8% glycerol by volume), contained 0.1 nM of each labelled promoter fragment, 200 ng salmon sperm DNA and varying amounts of the maltose-binding-PraR fusion protein (MBP-PraR) as described previously (Frederix *et al*., 2011). As indicated in Fig. S2, different amounts of CinS-His_5_ purified as described previously (Frederix *et al*., 2011) were added after the addition of 250 nM MBP-PraR.

### RNA purification and microarrays

Three independent *Rhizobium* cultures were grown in AMS minimal medium to exponential phase (OD_600_ 0.7–0.8). Samples (12 ml) were rapidly mixed with 24 ml RNAlater (Qiagen) and stored until further use. RNA was purified from *Rhizobium* cultures using the RNeasy Mini kit (Qiagen) as described in the manufacturer's instructions. Labelling and microarray analysis was performed as described previously using custom-designed arrays with unique 70-mer oligonucleotides representing 7344 genes of *R.l. viciae* strain 3841 as described previously (Karunakaran *et al*., 2009). Duplicates or triplicates of the 70mers were printed on the arrays in a random pattern such that each array included technical replicates and data were analysed using Genespring 7.2 (Silicon Genetics, Redwood, CA). After subtracting local background values from the intensity of each spot, a Lowess normalization was applied. Dye swaps were done on different biological repeats and the normalized expression ratios were calculated as described previously (Karunakaran *et al*., 2009).

### Radioactivity and bacterial count-based root attachment assays

Pea seeds were surface-sterilized by washing with 70% ethanol and incubating in 1% hypochlorite for 5 min then germinated on water agar plates in the dark at room temperature for 4–5 days. Bacteria were grown overnight in Y mannitol MOPS containing 185 kBq of ^3^H-leucine to OD_600_ 0.5. Cells were pelleted washed twice and then resuspended in an equal volume of 25 mM phosphate buffer pH 6.0. Aliquots were counted showing that 23.7 kBq had been incorporated and that there was less than 2% difference of incorporation in the two samples. Aliquots (100 μl) were added to 50 ml Falcon tubes containing 10 ml FP medium and 10 pea roots (2 cm lengths from the root tip). Tubes were incubated on a rocking platform for 2 h at room temperature. The roots were then washed three times with 20 ml of FP medium. The roots were then put into separate scintillation vials containing HiSafe2 scintillation fluid (Perkin Elmer) and counted in a Perkin Elmer Scintillation Counter.

In parallel experiments performed without radioactivity labelling, after incubation with bacteria and washing of the roots, attached bacteria were released by extensive vortexing (30 min) of the roots in phosphate buffer containing 0.5 mM EDTA and the released bacteria were counted by plating.

### Luminescence-based root attachment arrays

A stably inherited plasmid conferring luminescence was constructed using the pJP2 replicon (Prell *et al*., 2002). To generate a fragment with the *luxCDABE* operon containing appropriate upstream cloning sites, pBlueLux (Brackman *et al*., 2008) was first linearized by digestion with XhoI and EcoRI and then recircularized, ligating in a linker made by annealing the oligonucleotides 5′- TCGAGGGTACCCTCGAGGGATCCGTTTAAACG-3′ and 5′-AATTCGTTTAAACGGATCCCTCGAGGGTACCC-3′); this introduced KpnI, XhoI, BamHI, PmeI and EcoRI sites upstream of *luxC* and there is a PstI site downstream of *luxE*. The 6 kb KpnI–PstI fragment containing the *luxCDABE* operon was then cloned into KpnI- and PstI-digested pJP2 to generate pIJ11268, in which the β-glucuronaidase has been replaced by the *lux* operon with unique KpnI, XhoI, BamHI and PmeI sites upstream of *luxC* (Fig. S3). To generate pIJ11282, in which the *lux* operon is expressed under the control of the *nptII* promoter from Tn*5* (Fig. S3), the *nptII* promoter was amplified as a 400 bp fragment (using the primers 5′-TTTGGTACCAGGCCTGAATCGCCCCATC-3′ and 5′-CTTCGAATTCGAGCTCCCGGGTAC-3′, cut with KpnI and EcoRI and cloned into the same sites upstream of *luxC* in pIJ11268.

Plasmid pIJ11282 was then transferred by conjugation into *R.l. viciae* strains and these strains were grown, resuspended in phosphate buffer and added to pea roots as described above. The roots were then washed as described above and attached bacteria were measured by luminescence visualized using a NightOWL LB 983 *in vivo* Imaging System (Berthold Technologies, Bad Wildbad Germany); luminescence from individual roots was quantified using an FB12 Luminometer (Berthold Technologies). Luminescence is scored as relative light units; data shown in individual figures were all collected under identical conditions, and so are directly comparable. The RLU values may vary between experiments but the ratios of the signals remained very similar within experiments.
