# Supplementary material for: Mutation of praR in Rhizobium leguminosarum enhances root biofilms, improving nodulation competitiveness by increased expression of attachment proteins
Source: Mol Microbiol. 2014 Jul 2;93(3):464–78. doi: 10.1111/mmi.12670 (PMC4149787; doi:10.1111/mmi.12670)
Supplement: Supplementary file 1 — Supporting information [file mmi0093-0464-SD1.pdf]

Mutation of *praR* in *Rhizobium leguminosarum* enhances root biofilms, improving nodulation competitiveness by increased expression of attachment proteins

Marijke Frederix[*%], Anne Edwards[*], Anna Swiderska[§], Andrew Stanger, Ramakrishnan Karunakaran, Alan Williams[#], Pamela Abbruscato[+], Maria Sanchez-Contreras, Philip S. Poole[†], J. Allan Downie[‡]

Department of Molecular Microbiology, John Innes Centre, Norwich Research Park, Norwich, NR4 7UH, UK

SUPPLEMENTARY MATERIAL:

Supplementary Table S1. Other Plasmids Used

**Plasmid**	**Description**	**Reference**
pBlueLux	Plasmid containing *luxCDABE*	(Brackman *et al.*, 2008)
pGEM T-easy	Vector for T/A cloning, Amp^R	Promega
pIJ773	Plasmid containing *aac(3)IV* gene	(Gust *et al.*, 2004)
pJP2	Broad host range plasmid Tet^R	(Prell *et al.*, 2002)
pJQ173	For exchanging Kan^R to Spec^R in Tn5	(Quandt *et al.*, 2004)
pJQ175	For exchanging Kan^R to Gent^R in Tn5	(Quandt *et al.*, 2004)
pJQ200KS	Suicide vector	(Quandt and Hynes, 1993)
pIJ773	Plasmid containing *aac(3)IV* gene	(Gust *et al.*, 2004)
pK19mob	Integrative plasmid Kan^R	(Schäfer *et al.*, 1994)
pMP45Ω	Plasmid containing Spec^R	(Prentki and Krisch, 1984)
pMP220	Broad host range *lac*Z reporter Tet^R	(Spaink *et al.*, 1987)
pRK2013	Helper plasmid for conjugation Kan^R	(Ditta *et al.*, 1980)
pRU1156	Broad host range *gus* reporter Tet^R	(Karunakaran *et al.*, 2005)

Supplementary Table S2. Primers Used

Primer	Sequence	Size of amplified Fragment (bp)
**A.Promoter**		
P*cadA*F	AAGCTTGCATCGACCCCTTC	813
p*cadA*R	ATCGTAAGCTTCGAACTCAG	
p*cadB*F	AAGCTTGCGTAGTCGCCTATCTCAAG	874
p*cadB*R	GTCGTAAGCTTCGAACTCGG	
p*gmsA*F	TTTTGAATTCTTACTCCACAACAAAGGTGCC	222
p*gmsA*R	TTTTCTGCAGGGCTGGACGACAGACATCC	
p*plyA*F	TTTTAAGCTTCATGGTAATTATTGCGTTAATATA	562
p*plyA*R	TTTAAGCTTGTTCTGATTTGATCCTGTTGCC	
p*plyC*F	TTTAAGCTTAAAATTATGCAGCAATTC	532
p*plyC*R	TTTAAGCTTGGTCGGAGCCAGTCGCTGAGTT	
p*praR*F	GAGGTGCTGCTGATTTTGATTG	356
p*praR*R	GTATTTCTGGATCTGCTGGAAGGTG	
p*pssA*F	TTTTGAATTCCTTCATCTCCTGGGGTGG	667
p*pssA*R	TTTTCTGCAGCAATGGTTAACCCTGTCACCAG	
p*rapA2*F	TTTTGAATTCCTGCGCCTTTTC	777
p*rapA2*R	TTTTCTGCAGTTGTTTATTAGCGGGTCAATTTC	
p*rapB*F	TTTAAGCTTCTCGAACGCGGCAGACTG	542
p*rapB*R	TTTAAGCTTGGTATACATCACCT	
p*rapC*F	TTTTGAATTCGATGCGGCGAAG	311
p*rapC*R	TTTTCTGCAGAAGCGCTGCTGA	
pRL0149F	TTTTGAATTCCGGAATCGTAAAGC	478
pRL0149R	TTTTGAATTCGAAAGTCAGTCCGAGTGCTTC	
p*rosR*F	TTTTGAATTCGGCAAATGGCAACACGC	862
p*rosR*R	TTTTCTGCAGATCCACAAGCAGCTCCG	
**B. Mutant Isolation**		
*rapA2*F	TTTTGGTACCTTTCGAGTTCTCCTTGTCT	
*rapA2*R	AAAAACTAGTAAGAATTATGCCGCTTTG	
cadA	CTGCAAGTCCCACCTCGGTG	
cadB	CCGAGTTCGAATCTTACGACG	
RL0149	CTGATGCACTTCATCCCCCAATAC	
plyB	TTCTCGGGACCAAGGCAG	
rapB	CGCTCCCTTTACTTGAACAG	
*rapC*F	TTTTAAGCTTAACTTCTTCGATGGCGAGCG	
*rapC*R	TTTTTCTAGACAGGAAGAGCGTGCCAGC	
rosR	TCAATTCCAAATACGAGGCG	
**C. Other**		
specF	TTTTTCCGGAGGTGATTGATTGAGCAAGC	
specR	TTTTTCCGGACGGTGATTGATTGAGCAAG	
Tn5	GAACGTTACCATGTTAGGAGGT	
papra	TTTGTTGGATCCACCAAGGAAAGTCTACACGAAC	
aac5	CATGGATCCTCATGAGCTCAGCCAATCGAC	

Introduced restriction sites are underlined. Amplified fragment sizes include the full primers.

praR	CGC	TTGCAA	CGT
RL0149	ACT	TTGCAA	CTG
rosR	GAA	TTGCAA	TCG
rapA2	GAA	TTGCAA	CGG
rapB	CGG	TTGCCA	GAT
rapC	TGC	TTGCAA	AAC
rhiR	CGC	TTGAAA	CCA
Oligo	TCT	TTACAA	CCC

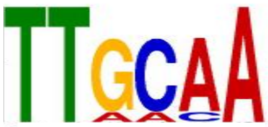

Supplementary Fig. S1 Predicted PraR-binding motif. The sequences of the PraR-binding promoter fragments upstream of the *rhiR*, *rapA2, rapB, rapC, rosR, praR and* RL0149 genes were analysed using MEME (Bailey and Elkan, 1994) for potential conserved sequences that were also present within a synthetic PraR-binding oligonucleotide (Frederix *et al.*, 2011). A conserved motif was identified and the predicted PraR binding sequences on each fragment are shown in colour along with three flanking nucleotides

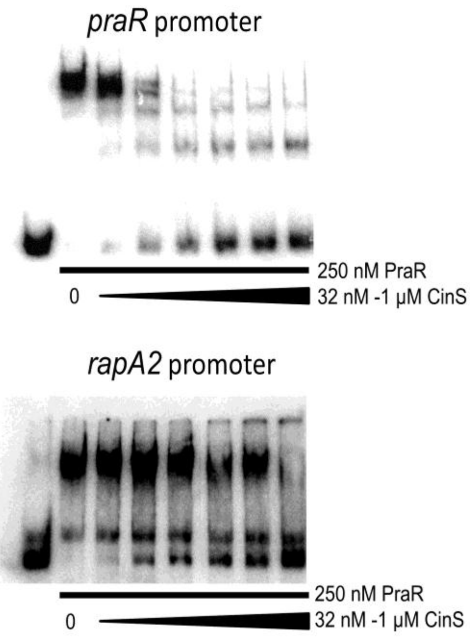

Supplementary Fig.S2 *In vitro* analysis of effects of CinS on PraR binding to the *praR* and *rapA2* promoters*.* Radioactively-labelled promoters of the genes indicated were incubated with 0 (leftmost lanes) or 250 nM purified PraR maltose-binding protein fusion. Purified CinS protein was added at different concentrations (0, 32 nM, 64 nM, 125 nM 250 nM, 500 nM and 1000 nM). After the reactions the samples were separated by non-denaturing gel electrophoresis and the radioactively-labelled bands were imaged using a phosphorimager as described previously (Frederix *et al.,* 2011).

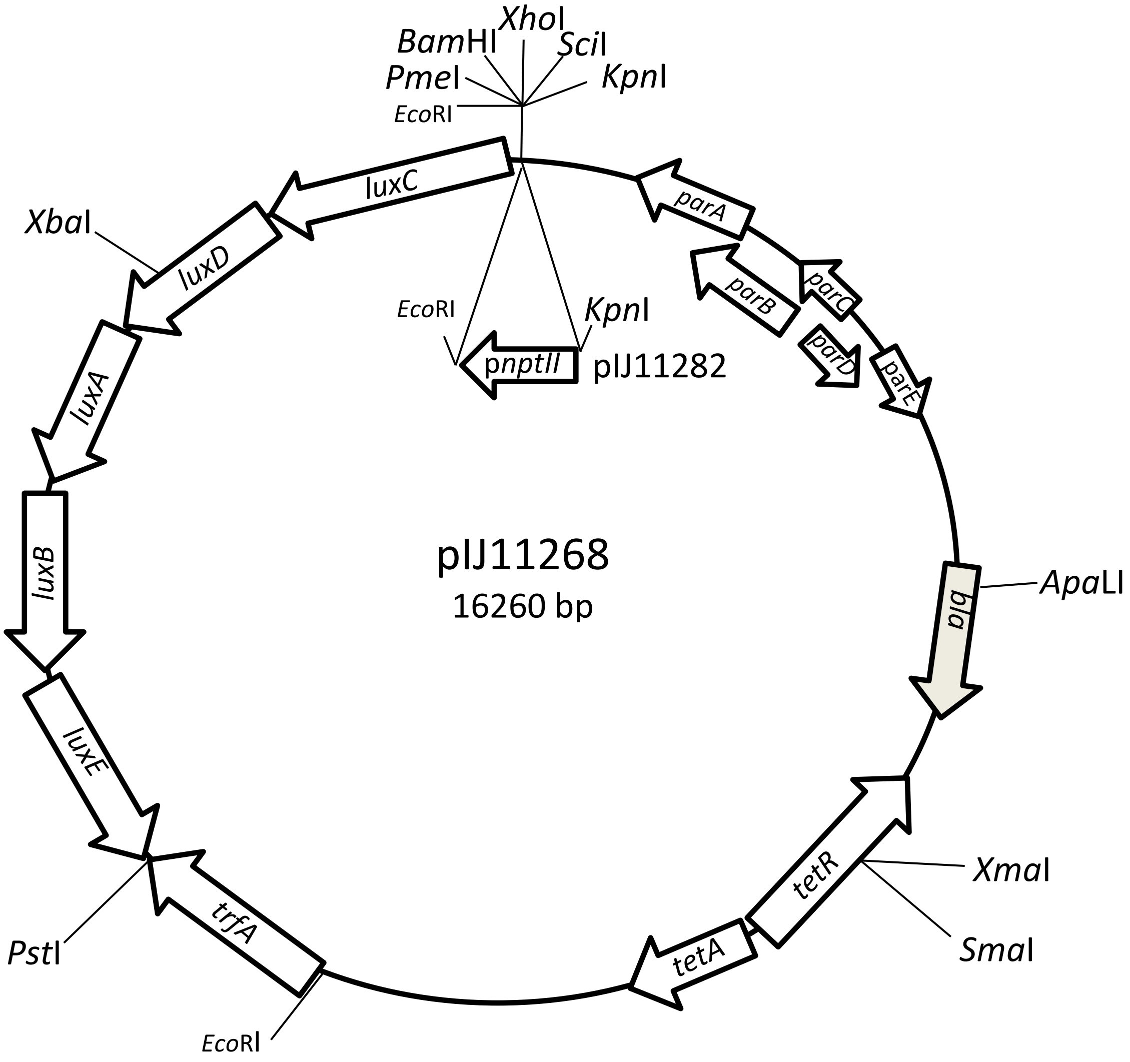

Supplementary Fig. S3. Map of the *lux* plasmid pIJ11268 and the derivative pIJ11282 constitutively expressing *luxCDABE*. pIJ11268 was made from the stably-inherited tetracycline-resistant plasmid pJP2 (that carries the plasmid partition genes *parABCDE),* by cloning in the *luxCDABE* operon on a *Kpn*I-*Pst*I fragment with a polylinker upstream of *luxC*. pIJ11268 lacks a promoter and so pIJ11282 was made by PCR-amplifying the *nptII* promoter from Tn*5* and cloning it using *Eco*RI and *Kpn*I sites introduced into the PCR primers. Unique restriction sites are shown in bold.

References

Bailey, T.L., and Elkan, C. (1994) Fitting a mixture model by expectation maximisation to discover motifs in biopolymers. Proc Int Conf Intell Syst Mol Biol **2**: 28–36.

Brackman, G., Defoirdt, T., Miyamoto, C., Bossier, P., Van Calenbergh, S., Nelis, H.*, et al.* (2008) Cinnamaldehyde and cinnamaldehyde derivatives reduce virulence in *Vibrio* spp. by decreasing the DNA-binding activity of the quorum sensing response regulator LuxR. *BMC Microbiol* **8:** 149.

Ditta, G., Stanfield, S., Corbin, D., and Helinski, D.R. (1980) Broad host range DNA cloning system for gram-negative bacteria: construction of a gene bank of *Rhizobium meliloti*. *Proc Natl Acad Sci U S A* **77:** 7347-7351.

Frederix, M., Edwards, A., McAnulla, C., and Downie, J.A. (2011) Co-ordination of quorum-sensing regulation in *Rhizobium leguminosarum* by induction of an anti-repressor. *Mol Microbiol* **81:** 994-1007.

Gust, B., Chandra, G., Jakimowicz, D., Yuqing, T., Bruton, C.J., and Chater, K.F. (2004) Lambda red-mediated genetic manipulation of antibiotic-producing *Streptomyces*. *Adv Appl Microbiol* **54:** 107-128.

Karunakaran, R., Mauchline, T.H., Hosie, A.H., and Poole, P.S. (2005) A family of promoter probe vectors incorporating autofluorescent and chromogenic reporter proteins for studying gene expression in Gram-negative bacteria. *Microbiology* **151:** 3249-3256.

Prell, J., Boesten, B., Poole, P., and Priefer, U.B. (2002) The Rhizobium leguminosarum bv. viciae VF39 gamma-aminobutyrate (GABA) aminotransferase gene (gabT) is induced by GABA and highly expressed in bacteroids. *Microbiology* **148:** 615-623.

Prentki, P., and Krisch, H.M. (1984) *In vitro* insertional mutagenesis with a selectable DNA fragment. *Gene* **29:** 303-313.

Quandt, J., Clark, R.G., Venter, A.P., Clark, S.R., Twelker, S., and Hynes, M.F. (2004) Modified RP4 and Tn5-Mob derivatives for facilitated manipulation of large plasmids in Gram-negative bacteria. *Plasmid* **52:** 1-12.

Quandt, J., and Hynes, M.F. (1993) Versatile suicide vectors which allow direct selection for gene replacement in gram-negative bacteria. *Gene* 127: 15-21.

Schäfer, A., Tauch, A., Jäager, W., Kalinowski, J., Thierbach, G., and Pühler, A. (1994) Small mobilizable multi-purpose cloning vectors derived from the *Escherichia coli* plasmids pK18 and pK19: selection of defined deletions in the chromosome of *Corynebacterium glutamicum*. *Gene* **145:** 69-73.

Spaink, H., Okker, R.J., Wijffelman, C.A., Pees, E., and Lugtenberg, B. (1987) Promoters in the nodulation region of the *Rhizobium leguminosarum* Sym plasmid pRL1JI. . *Plant Mol Biol* **9:** 27-39.
